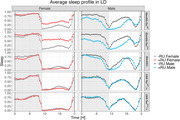# Expression of human tau in adult *Drosophila melanogaster* causes a sexually dimorphic sleep phenotype

**DOI:** 10.1002/alz.088411

**Published:** 2025-01-03

**Authors:** Edmond N Mouofo, James Catterson, Patrik Verstreken, Tara L. Spires‐Jones

**Affiliations:** ^1^ UK Dementia Research Insitute at The University of Edinburgh, Edinburgh United Kingdom; ^2^ UK Dementia Research Insitute at The University of Edinburgh, Edinburgh, United Kingdom, Edinburgh United Kingdom; ^3^ Department of Neurosciences, Leuven Brain Institute, VIB Center for Brain & Disease Research, KU Leuven, Leuven Belgium; ^4^ UK Dementia Research Institute at the University of Edinburgh, Edinburgh, Scotland United Kingdom

## Abstract

**Background:**

Alzheimer’s disease (AD) is the primary cause of dementia, characterized by early amyloid beta accumulation, subsequent tau pathology, and eventually synaptic and neuronal loss. Sleep disturbances, a clinical phenotype in AD, are linked to amyloid beta and impaired protein clearance. However, the influence of tau pathology on sleep is less explored. Utilizing *Drosophila melanogaster*, we investigate the effects of adult‐onset tau expression on sleep. This study addresses a knowledge gap regarding the impact of tauopathy on sleep‐related changes contributing to our understanding of AD progression.

**Method:**

We investigated the impact of adult‐onset pan‐neuronal expression of wildtype tau and pseudo‐phosphorylated tau E14 in male and female *Drosophila*, induced by the gene‐switch drug RU 486 (mifepristone). The flies were exposed to the inducer added to their food from 2‐3 days post eclosion. Utilizing the *Drosophila* Activity Monitor (DAMs), we monitored activity and sleep patterns following expression for 2 weeks (n>47 flies per study group) and 4 weeks (n>17 flies per study group). Subsequently, we analysed and plotted data on activity and sleep. Additionally, at the 4‐week mark, we conducted immunohistochemistry on the flies to assess neurodegeneration, and performed lifespan assays.

**Result:**

Results consistently revealed that flies expressing tau exhibited a shorter median lifespan compared to the control group. Unexpectedly, our gene‐switch control flies displayed a marked heightened activity leading to confounding behavioural effects in our analysis. Despite the activity confounds in one control line, neither the genetic controls nor the RU inducer affected sleep phenotypes, allowing investigation of sleep disruptions due to tau expression. We observe female but not male flies expressing tau have increased day sleep bouts length compared to controls. The pseudrophosphorylated E14 tau expression exacerbated/accelerated the phenotype compared to wild‐type tau. Immunohistochemistry showed elevated vacuoles in tau‐expressing flies indicating neurodegeneration which likely caused the sleep phenotype.

**Conclusion:**

Our results indicate that pathological tau expression in *Drosophila melanogaster* causes neurodegeneration and increase sleep in females. These results indicate that pathological tau may directly contribute to sleep disruption in humans and provides a new model system to further our understanding of the role of tau in neurodegeneration.